# Sub-wavelength Unidirectional Antenna Realized by Stacked Spoof Localized Surface Plasmon Resonators

**DOI:** 10.1038/srep29773

**Published:** 2016-07-13

**Authors:** Feifei Qin, Qiang Zhang, Jun-Jun Xiao

**Affiliations:** 1 College of Electronic and Information Engineering, Shenzhen Graduate School, Harbin Institute of Technology, Xili, Shenzhen 518055, China

## Abstract

The use of resonant structures to control scattering strength and directionality is of importance in various electromagnetic systems. Here we propose and demonstrate sub-wavelength unidirectional scattering by two nearby spoof localized surface plasmon resonators for microwave. The principle is that metal surfaces corrugated by grooves can support magnetic dipolar modes, as well as electric dipolar modes. The resonance is essentially dictated by the geometric parameter of the structure, enabling extremely high degrees of freedom for tuning the scattering properties of the resonator. Particularly, by adjusting the thickness of the resonators, we can make the magnetic dipole mode of one resonator have nearly the same resonant frequency with that of the electric dipole mode of the other resonator. We show that nearly zero backscattering happens when the distance between the two resonators is subwavelenght but larger than a certain value, otherwise strong vertical coupling and mode splitting occur. The results can be extended to other frequency bands and might find application in unique resonant devices as a radio frequency (RF) antenna, filter and metasurface.

Optical nanoantennas have drawn much attention in recent years^1^,^2^,^3^,^4^,^5^, for their ability to emit and collect electromagnetic (EM) waves^6^. Moreover, the optical antennas show promise in targeted medicine application^7^,^8^ and photovoltaics^9^. In many of these applications, emission and scattering direction control is of crucial importance. So far, different kinds of nanoantennas, including Yagi-Uda^10^,^11^ and patch antennas^12^, have been proposed and have demonstrated the ability to obtain strong directionality in antenna characteristics. The uni-directionality derives from a configuration of locally driven feed elements coupled with several parasitic director elements in the far field. However, such array antennas are complex to design and have a relatively large spatial footprint in the RF and terahertz region.

However, Kerker *et al*.^13^ have noted that, by overlapping electric and magnetic dipolar resonances of the same strength in the same spectrum window, the scattered field of a Mie scatter can have constructive (destructive) interferences in the forward (backward) direction. Such a scheme is now referred to as the Kerker condition. It is often utilized to design the unidirectional plasmonic or high refractive index dielectric optical antenna in the optical regime. However, using plasmonic particles in RF will result in a barrier in which metals behave similarly to perfect electric conductors (PECs) in this spectrum regime, with no occurrence of fundamental or dramatic surface plasmon resonance. To exploit the exciting properties of surface plasmons in RF or terahertz, Pendry *et al*.^14^,^15^ first proposed the concept of spoof surface plasmon polaritons (SSPPs), which enables the possibility of using sub-wavelength resonant structures. By the mechanism of SSPPs, spoof localized surface plasmon (SLSP) resonators were realized by texturing the circumference of a PEC or quasi-PEC disk^16^,^17^ and these types of SLSP resonators have been explored both theoretically and experimentally. It was shown that both electric dipole (ED) and magnetic dipole (MD) resonances can be supported and engineered to be of comparable strength^18^. Therefore, they can be used as sub-wavelength RF antenna and have prominent potential in building integrated microwave device. Moreover, SLSP resonators have a greater degree of freedom for tuning resonance properties by adjusting the geometry parameters including radius, thickness, and surface textures profile^19^,^20^, in addition to the filling medium.

In this work, we studied the EM response by combining two such SLSP resonators. It was demonstrated that, by carefully designing these resonators, a unidirectional sub-wavelength antenna can be achieved. The design is fundamentally based on the Kerker condition, and zero backscattering is shown for a particular geometrical configuration that would be easily realized in experiment.

## Results

### ED and MD of the same frequency in different SLSP resonators

We start by considering the first Kerker condition^13^, in which an antenna exhibits comparable electric and magnetic multipole scattering coefficients and the scattering fields destructively interfere in the backward propagating direction^21^. The structure is much smaller compared with the resonance wavelength and is illuminated by a plane wave propagating along the positive **x** axis. Then it is possible to assume that the induced EDs and MDs are superimposed in real space at the origin of the coordinate system. For a *y*-oriented ED *p*_*y*_ and a *z*-oriented MD *m*_*z*_, the radiated field of the structure in the backward direction reads as^22^,^23^,^24^,^25^ (see also the Method)


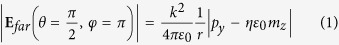


where *r* is the length of the radius-vector from the dipole location (coordinate origin) to the observation point, *k* = *ω*/*c* is the wave vector in the vacuum, 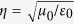 denotes the free space impedance; and *θ* and *φ* are the angles with respect to the *z –*and *x-*axes, respectively. According to Eq. (1), zero backscattering can be achieved on the *xy* -plane if one gets


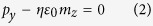


**Figure 1:**
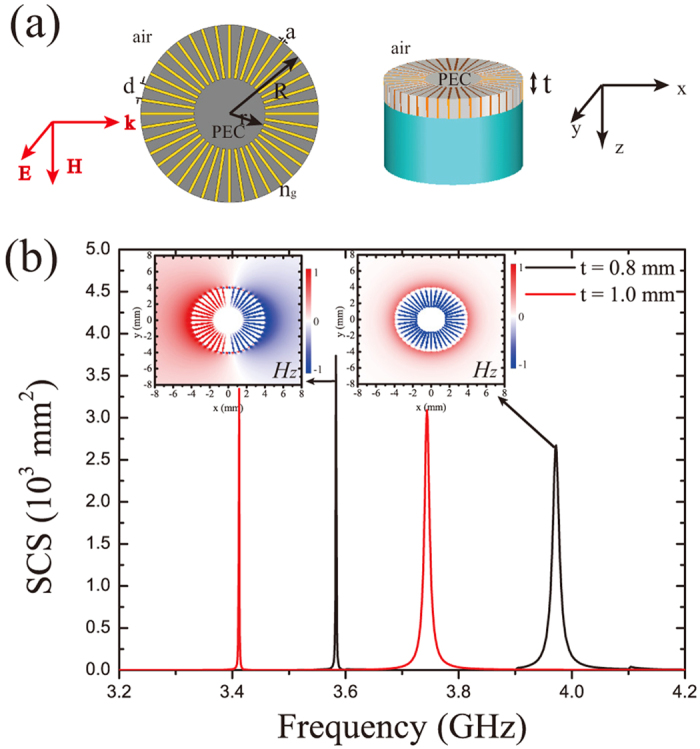
Geometry and resonant properties of magnetic SLSP resonators corrugated with dielectric grooves. (**a**) The geometry and scattering configration. (**b**) SCS for thickness *t* = 0.8 mm (black line) and *t* = 1.0 mm (red line). The other parameters are: *R* = 4 mm, *r* = 1.6 mm, *d* = 0.628 mm, *a* = 0.01256 mm and *n*_*g*_ = 12. Left (and right) insets: Near-field distribution of the electric (and magnetic) dipole resonances for a disk of thickness *t* = 0.8 mm.

Equation (2) provides a general guideline to design nanoantennas with zero backscattering. As such, we would show how to use SLSP resonators to build a zero backscattering antenna based on it. Firstly, we would revisit the electromagnetic responses of a cylindrical structure with overall dimensions that are less than the free space wavelength. Figure 1(a) shows the cross section layout and the whole structure of a three-dimensional SLSP resonator, which consists of periodic grooves of parallel metallic walls with an inner radius *r* = 1.6 mm and an outer radius *R* = 4 mm. The periodicity and groove width are *d* = 0.628 mm and *a* = 0.01256 mm, respectively. The grooves are filled with a dielectric material of refractive index *n*_*g*_ = 12 and the resonator is supported by a substrate of thickness *t*_0_ = 4 mm made of dielectric medium of refractive index *n*_0_ = 1.5. At sufficiently low frequencies, we can regard the cylinder approximately as a PEC. The resonance condition for the fundamental modes of a 2D SLSP resonator is given by the following transcendental equation^18^:


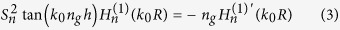


where 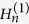 is the Hankel function of the first kind, 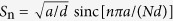, *n* the mode number; *h* = *R* − *r* and 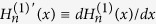.

Figure 1(b) shows the scattering spectrum of two individual SLSP resonators with thicknesses *t* = 0.8 mm and 1.0 mm, respectively, calculated by the finite element method solver COMSOL Multiphysics^26^. Here and in the following sections, an incoming TM-polarized plane wave (magnetic field 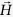 along the *z* -direction) propagating along the +*x* axis and with wave number *k*_0_ = *ω*/*c* is considered. We can see in Fig. 1(b) that the scattering cross section (SCS) spectra for resonators of different thickness both exhibit two distinct peaks. By examining the corresponding field patterns of these resonances, it is clear that the low-frequency resonance corresponds to an ED mode pointing along the *y* axis [see the left inset of Fig. 1(b)] and the one at the higher frequency is a *z* -axis oriented MD mode [see the right inset of Fig. 1(b)]. The SCSs and mode patterns show that the designed corrugated disks of finite thickness support a magnetic SLSP at a different frequency, with respect to the electric SLSP. Furthermore, both the two kinds of resonances shift when the resonator size and thickness vary.

**Figure 2:**
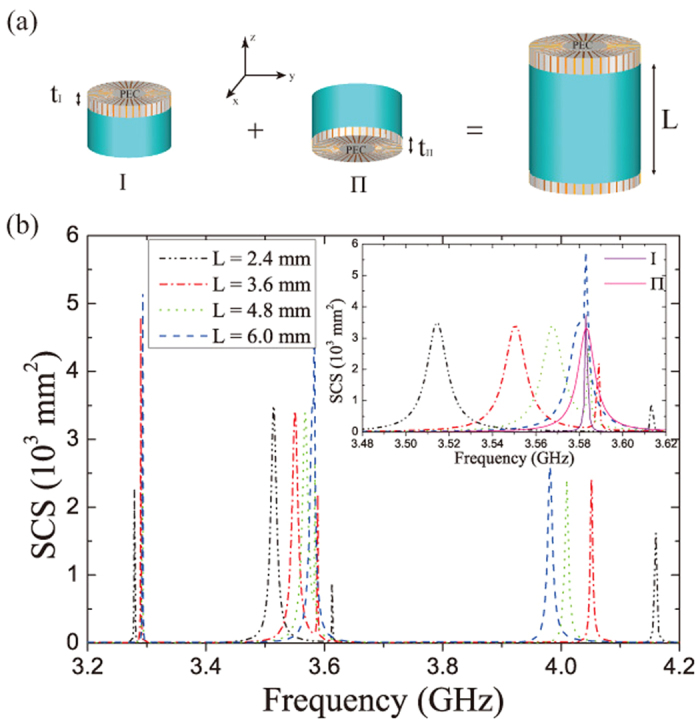
Geometry and resonant properties of composite SLSP resonators. (**a**) Schematic of the composite resonator. (**b**) SCS for the composite SLSP resonator with different separations *L* = 2.4 mm, 3.6 mm, 4.8 mm, and 6.0 mm). Left inset: SCS spectrum of the individual sub-resonator I and II.

With respect to such characteristics, we then placed two SLSP resonators side by side, in an attempt to design a unidirectional zero backscattering antenna, as schematically shown in Fig. 2(a). The two SLSP resonators were separated by a common dielectric substrate (for example F4B dielectric board) of thickness *L*. By adjusting the thickness *t*_I_ and *t*_II_ of the two SLSP resonators, we found that the MD of resonator Π overlapped well with the ED of resonator Ι at *f* = 3.583 GHz in the spectrum [see the inset of Fig. 2(b), notice that the purple line corresponding to sub-resonator Ι and the pink line corresponding to sub-resonator Π], when the thickness of the resonator Ι is *t*_I_ = 0.8 mm and that of resonator Π is *t*_Π_ = 1.2 mm.

The distance between the two resonators is then set as *L* = 2.4 mm to 3.6 mm, 4.8 mm, and 6.0 mm successively, and the SCS spectra for the composite resonator [i.e., the combined structure in Fig. 2(a)] are shown in Fig. 2(b). It is clearly observed that each spectrum exhibits four major visible resonance peaks expect for the case of *L* = 6.0 mm. As *L* increased from 2.4 mm to 6.0 mm, the two peaks in the middle move closer to each other and finally converge into a single one. We stress that at this point, the size of the overall composite resonator is less than 10 mm, approximately one-tenth of the operating wavelength (~100 mm). It is reasonable to deduce that the spectral separation of the middle peaks would increase when the spacer thickness is decreased, because of the stronger interaction among the different resonance modes. However, the other two peaks at the low and high frequencies also shift, and the shifting strength decreases for increased separation. These observations indicate that a complex interaction between the two resonators and that all four modes in resonator I and resonator II might be involved. To explore further the interaction between the two SLSP resonators, we would separately examine the cases across the frequency merging point in detail.

### Vertical coupling and mode shifting

**Figure 3:**
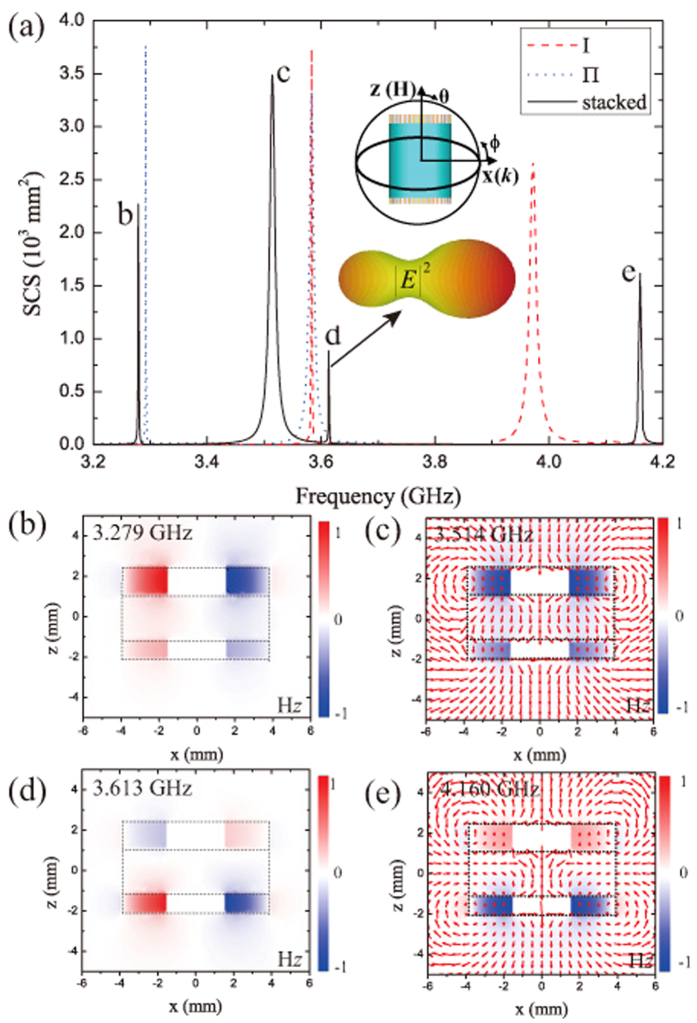
Mode interaction and hybridization in composite resonator. (**a**) SCS for the 3D composite SLSP resonator. The peaks in the composite resonator spectrum are marked by “b”, “c”, “d”, “e”, the red dashed line and blue dotted line correspond to the SCS for the sub-resonator Ι and for the sub-resonator Π. The top inset in (**a**) represents the 3D composite SLSP resonator with separation *L* = 2.4 mm and the bottom inset represent the radiation pattern of the third peak. (**b**–**e**) near field (*H*_*z*_) pattern for the frequency points “b”, “c”, “d”, and “e” labeled in (**a**). The arrows in (**c**,**e**) show the magnetic field line.

Figure 3(a) shows the SCS spectra for the composite SLSP resonator with the separation *L* = 2.4 mm (solid curve) and for the two sub-resonators (dashed and dotted curves, respectively). Obvious spectral shifts occurred among the four peaks for the composite SLSP resonator, with respect to those of the isolated resonators I and II. This is similar to the results reported in our previous work where two sub-resonators were essentially of separation *L* = 0^27^. The *z* -component magnetic fields in the *xz* -plane for the peaks [labeled by b, c, d and e in Fig. 3(a)] are shown in Fig. 3(b–e), respectively. Clearly, both the SLSP resonators at resonances *f* = 3.279 GHz [Fig. 3(b)] and *f* = 3.613 GHz [Fig. 3(d)] have ED characteristics. However, the fields are strongly localized and enhanced in one resonator (i.e., substructure I or II) and appear weaker in the other resonator. This means that the resonance basically comes from one of the two resonators. At the frequency *f* = 3.279 GHz, the fields at the top and bottom resonators are in-phase, whereas they are out-of-phase for the resonance at *f* = 3.613 GHz. For the second and the fourth peaks, we can recognize that both SLSP resonators are of MD mode by the vectorial plots in Fig. 3(c,e). Similar to the cases in Fig. 3(b,d), the fields are concentrated mostly on the top resonator at *f* = 3.514 GHz [Fig. 3(c)], while at the resonance *f* = 4.160 GHz, the bottom resonator is more intensively excited [Fig. 3(e)]. Again, they are, respectively, in-phase and out-of-phase in Fig. 3(c,e).

The resonance modes supported by a SLSP resonator are confined not only to the *xy* -plane, but also along the *z* -direction^28^, which therefore provides vertical near-field coupling. It is anticipated that these vertical coupling only occur between the ED mode (MD mode) of one resonator and ED (MD) of the other resonator, which can be explained quantitatively by the coupled mode theory^29^,^30^,^31^. Because of these vertical coupling mechanisms, the EDs and MDs supported by the SLSP resonators are separated from each other when the separation *L* is not large enough and cannot be superimposed in space at the same point. Thus, no significant unidirectional suppression of the scattering radiation could be achieved, and this has been confirmed by the 3D power pattern of the third peak [see the inset of Fig. 3(a)].

### Unidirectional scattering by the composite structure

**Figure 4:**
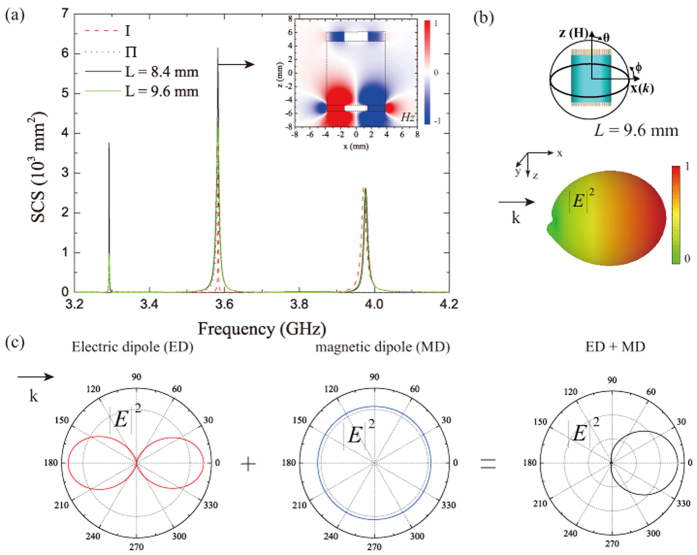
Unidirectional scattering by overlapped ED and MD resonances. (**a**) SCS for the 3D composite SLSP resonator with different separations (*L* = 8.4 mm, 9.6 mm.) the inset represents the near field (*H*_*z*_) pattern at 3.583 GHz. (**b**) The 3D radiation power pattern of the second peak. (**c**) Radiation power pattern in the *xy* plane for the two sub-resonators and the compositor SLSP resonator at 3.583 GHz.

As discussed in the previous section, when the separation *L* increases from 2.4 mm to 6.0 mm, the ED mode and the MD mode cannot superimpose in the same frequency because of vertical coupling. Therefore, no distinct unidirectional scattering occurs. Whereas, if we continue to increase the separation *L* beyond that, we see that the peaks of the ED mode and the MD mode eventually converge to one single peak at *f* ≈ 3.583 GHz and no longer vary for larger separation, as shown in Fig. 4(a). Meanwhile, the other two peaks for the composite SLSP resonator resemble those of the corresponding sub-resonators. The resonant *H*_*z*_ field is shown in the inset of Fig. 4(a) for *L* = 9.4 mm. It clearly shows that the top resonator is at a MD resonance mode and that the bottom resonator is in an excitation state of ED mode. More importantly, no strong vertical coupling is apparent between the two resonators in the near field. The 3D power pattern for the composite resonator at *f* = 3.583 GHz is shown in Fig. 4(b). Clearly, nearly zero backscattering phenomenon has been achieved. Figure 4(c) shows the power patterns of the *y* -oriented ED mode, the *z* -oriented MD mode and the combinational effects produced by the interference between them on the *xy* -plane. This actually illustrates the physical mechanism behind the first Kerker condition. Hence, we can conclude that when the separation between the two SLSP resonators is larger enough to prevent strong vertical near-field coupling and mode hybridization, it would be easy to engineer the composite SLSP resonator for zero backscattering. It is also possible to adjust the geometric parameters of the SLSP resonator to make a unidirectional antenna working in other frequency band, in view of the flexible and tunable properties of SLSP.

**Figure 5:**
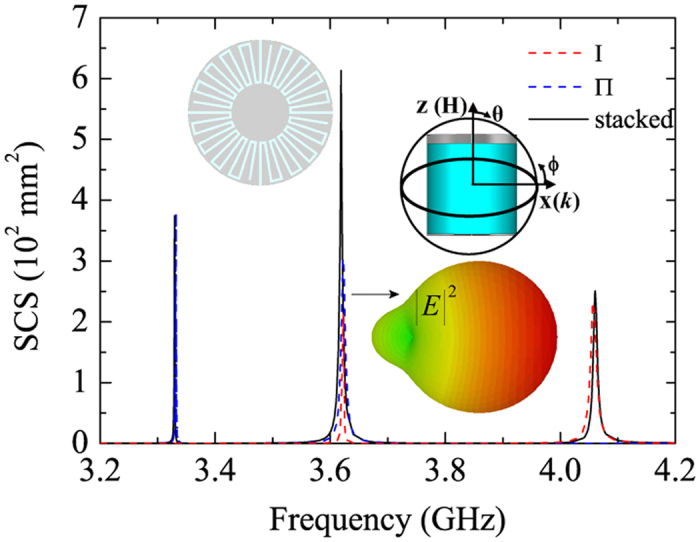
Unidirectional scattering by 3D composite SLSP resonator in PEC sub-wavelength structures without dielectric filling. The geometry is sketched as an inset in the left upper and the 3D radiation pattern of the second peak for the composite SLSP resonator is in the right bottom.

In summary, a few comments are in order. Whereas the high dielectric material in practice might be a hindrance, Equation (3) gives an indication of how to overcome. Because the resonance frequency of the sub-resonator relies mainly on *n*_*g*_*h*, we must increase the depth *h* of the grooves to decrease the dielectric function *n*_*g*_ of the filling material. But to keep the antenna sub-wavelength, one option has been proposed by using meander-shape grooves to increase their effective depth *h*_*m*_^18^. Here, we show this approach by replacing the resonator with PEC disks corrugated with meanders [see the left inset of Fig. 5]. The new geometric configuration consists of a cylinder radius *R* = 4 mm, textured with four meander-shaped grooves of effective depth *h*_*m*_ ≈ 2*πR*(*R* − *r*)/4*d* = 24 mm. The thicknesses of the substructure Ι and substructure Π are *t*_I_ = 0.8 mm and *t*_II_ = 0.146 mm, respectively. No high-refractivity material filling these grooves is required. The SCS spectra for the composite resonator with separation *L* = 8 mm and for the two individual sub-resonators are shown in Fig. 5. Remarkably, similar characteristics for the previous composite resonator of *n*_*g*_ = 12 are seen. The radiation power pattern for the second peak is shown as the right inset of Fig. 5, which clearly demonstrates nearly perfect suppression of backscattering.

## Discussion

In conclusion, we proposed an RF unidirectional antenna constructed by two SLSP resonators. We show that, by reasonable adjusting the geometric parameter of the composite SLSP resonators, the first Kerker condition can be satisfied. Here, we stress that the separation between the two SLSP resonators should be larger than a certain value to avoid ventral near-field coupling and mode hybridization between the two, in order to separate the ED mode and MD mode. Such findings provide an alternative way to design sub-wavelength unidirectional antenna in microwave and terahertz region.

## Method

### The first Kerker condition

The far field radiated by an electric dipole **p** and a magnetic dipole **m** in vacuum is given by


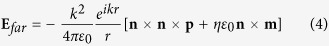


For the combination of a *y* -oriented ED **p** = (0, *p*_*y*_, 0) and a *z* -oriented MD **m** = (0, 0, *m*_*z*_), the electric field reads


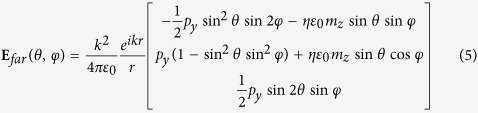


Considering the back-scattering (*θ* = *π*/2, *φ* = *π*) of the resonator that is illuminated by a plane wave propagating along the +*x* axis, one arrives at Eq. (1).

### Simulation method

The constituent material metal is highly conductive at the frequency of interest and can be simply treated as PEC (conductivity tends to infinity and electric field is zero at the inner boundary). Here, driven-solver was used by impinging a plan wave and the total SLSP resonators are surrounded by perfectly-matched layers to eliminate the artificial reflections. The scattering cross sections are obtained by integrating the normal scattered Poynting vector on a closed curve that encloses the whole composite resonator.

## Additional Information

**How to cite this article**: Qin, F. *et al*. Sub-wavelength Unidirectional Antenna Realized by Stacked Spoof Localized Surface Plasmon Resonators. *Sci. Rep.* **6**, 29773; doi: 10.1038/srep29773 (2016).
